# De-novo and genome-wide meta-analyses identify a risk haplotype for congenital sensorineural deafness in Dalmatian dogs

**DOI:** 10.1038/s41598-022-19535-4

**Published:** 2022-09-14

**Authors:** B. Haase, C. E. Willet, T. Chew, G. Samaha, G. Child, C. M. Wade

**Affiliations:** 1 Sydney School of Veterinary Science, Faculty of Science, The University of Sydney, Camperdown, NSW Australia; 2 Sydney Informatics Hub, Core Research Facility, The University of Sydney, Camperdown, NSW Australia; 3 University Veterinary Teaching Hospital, Sydney School of Veterinary Science, Faculty of Science, The University of Sydney, Camperdown, NSW Australia; 4 School of Life and Environmental Sciences, Faculty of Science, The University of Sydney, Camperdown, NSW Australia

**Keywords:** Genome informatics, Genome-wide association studies, Haplotypes, Heritable quantitative trait, Medical genetics

## Abstract

Congenital sensorineural deafness (CSD) has been reported to affect up to 30% of Dalmatian dogs world-wide and while unilaterally deaf dogs can live a close to normal life, dogs suffering bilateral deafness are frequently euthanized. Extreme-white coat patterning as encoded by the gene *Melanocyte Inducing Transcription Factor* (*MITF*) has long been postulated as the major risk factor for CSD in the Dalmatian breed. While attempts to identify causative risk variants associated with CSD have been numerous, no genome-wide association study has positively identified *MITF* as a risk locus for either bilateral or unilateral deafness in the Dalmatian breed to date. In this study, we identified an association with CSD on CFA20 in the vicinity of *MITF* within Australian Dalmatian dogs. Although not genome-wide significant, the association signal was validated by reanalysing publicly available data and merging the wider data resource with the local data to improve statistical power. The merged data, representing three major global populations of Dalmatian dogs, enabled us to identify a single, well-defined genome-wide significant risk haplotype for CSD. The haplotype was formed by three genome-wide significant associated markers (BICF2G630233852T>C, BICF2G630233861T>C, BICF2G630233888G>A) on CFA20 with 62% of bilaterally deaf dogs homozygous for the risk haplotype (CCA), while 30% of bilaterally deaf and 45% of hearing dogs carried one copy of the risk haplotype. Animals homozygous or heterozygous for the low-risk haplotype were less likely to be unilaterally deaf. While the association between the risk haplotype and deafness is incomplete, animals homozygous for the risk haplotype were 10-times more likely to be bilaterally deaf. Although the underlying causative variants are yet to be discovered, results from this study can now assist with reducing deafness in Dalmatian dogs.

## Introduction

Congenital sensorineural deafness (CSD, OMIA #000259-9615) in dogs presents a major challenge to breeders and owners, and frequently results in the euthanasia of bilaterally deaf dogs. The prevalence of CSD varies among dog breeds, with the highest prevalence reported in Dalmatians^1–7^. Strict selection of breeding animals has decreased the prevalence of CSD in Dalmatians in some countries, however, around one in three Dalmatians world-wide is still affected by hearing loss in either one or both ears^8–12^. The clinical and histopathological aspects of CSD have been thoroughly examined, and two main types have been described: the neuroepithelial type, and the cochleo-saccular type^13–15^. One difference in the phenotypic expression of the two types is that the neuroepithelial type is usually expressed bilaterally and the cochleo-saccular type can be either unilateral or bilateral^16,17^. While disease progression varies, deafness in both types is caused by the degeneration of hair cells, the stria vascularis and the vestibular membrane^16–18^. Current findings indicate that the absence of melanocytes in the inner ear is the main underlying predisposing factor and that the cochleo-saccular type is associated with skin and coat pigmentation^5,6,12,18–21^.

In the dog, extreme-white coat patterning caused by the *S* locus and encoded by the gene *Melanocyte Inducing Transcription Factor* (*MITF*) has long been postulated as a major risk factor for CSD^1,22–25^. The accepted implication has been that extreme-white dogs are equally at risk for CSD by genotype, and that differential phenotypic expression is random. Extreme-white is a breed-hallmark trait of the Dalmatian dog breed, although varying expression of patching and blue eyes suggest that genetic variation exists at the *MITF* locus^26^. Dalmatians expressing pigmented patches on their head and/or body appear to have a reduced deafness risk, while the risk increases with the presence of one or two blue eyes^1,4,5,8,12,16^. Given our current understanding of the phenotypic effects of the gene *MITF*, both observations support the potential role of *MITF* in the variable expressivity of CSD.

Despite the clear phenotypic description of CSD, the high prevalence in some breeds, and numerous efforts to uncover the genetic basis of canine deafness, few candidates for causative variants have been identified so far, with none in the Dalmatian dog^27–31^. Attempts to identify causative risk variants associated with CSD in Dalmatian dogs have been numerous^25,30,32–38^. Most recently, a genome-wide association study (GWAS) that included 304 Dalmatian dogs with sensory phenotypes identified strong associations with bilateral deafness on CFA23, CFA30, CFA37 and CFA38^39^. In contrast, an earlier GWAS identified a range of association signals on alternative chromosomes (CFA6, CFA14, CFA27, CFA29 and CFA31)^35^. Interestingly, none of the GWAS conducted to date in the Dalmatian breed have positively identified *MITF* as a risk locus for either bilateral or unilateral deafness. There is inconsistency of associated loci between studies on the same breed, and this suggests that current analyses are either underpowered; the trait is truly complex; or that other factors such as differing phenotyping approaches and population effects are hampering the analyses. Furthermore, studies commonly apply brainstem auditory evoked response (BAER) testing to assess the clinical expression of deafness. BAER results can be obtained using a variety of equipment types and operators. Animals may be awake or sedated when the hearing is assessed. Varying clinical phenotyping conditions and techniques may lead to inaccurate clinical assessments that might particularly affect unilateral versus normal phenotypes, since these animals remain responsive to sound.

Two canine reference assemblies (Broad/canFam2, hereafter referred to as canFam2 and Broad CanFam3.1/canFam3, hereafter referred to as canFam3) include a structural mis-assembly of the *MITF* gene. This mis-assembly not only affects the placement of exon 1 of the *MITF-A* isoform but also the gene orientation relative to surrounding genes^40^. Analysis of the region is further complicated by the existence of retrotransposed pseudogenes of the *MITF-M* and *MITF-A* isoforms, with the *MITF-A* retrocopy located on the Y chromosome^40,41^. Array markers in recent commercial genotyping arrays (e.g. Illumina Canine 220 K array) include erroneous variants from the *MITF-A* pseudogene, including the presence of two putative mis-sense mutations (rs851676581 and rs851603213)^40^. While these factors together hamper the assessment of linkage-disequilibrium (LD) and haplotype throughout the *MITF* region, there is also the possibility that the retrotransposon itself or other genes located in the genomic region identified may affect the deafness phenotype^40^.

In this study, a GWAS of CSD using Australian Dalmatian dogs with well characterised hearing phenotypes is performed. Results are validated with public-domain data of independent Dalmatian populations from the UK and the US to improve statistical power to overcome random error associated with population effects and phenotyping.

## Results

### Genome-wide association analysis

The analysis considered 145 Dalmatians (86 hearing, 33 unilateral deaf and 26 bilateral deaf) from Australia, with hearing phenotypes available. After quality filtering, a total of 78,830 variants were included in the analysis. No individual marker reached genome-wide significance after multiple-test correction by Bonferroni (Table S2). The most significantly associated marker was BICF2G630233861 T>C (CFA20:22045960) P_raw_ = 8.44E − 06 (canFam3), with three of the top 10 markers located near or within the *MITF* gene. Of the remaining top 10 markers, one was located on CFA9, four on CFA34, one on CFA36 and one on CFA39. None of the top associated markers from this analysis validated previously identified associated loci (Table 1).

**Table 1:** Previously reported associated markers and results from this study.

SNP	CFA	Position (bp)	Original analysis		Meta-analysis (quantitative)	Meta-analysis (CMH)
			Cohort of origin	p-value	p-value	p-value
BICF2P176848	2	13786700	Kluth et al.	7.08E − 07	7.10E − 01	9.70E − 01
BICF2P590845	6	68927940	Kluth et al.	2.09E − 06	9.00E − 01	5.70E − 01
TIGRP2P83893_RS8732055	6	45474835	Kluth et al.	8.13E − 06		
BICF2G630529431	14	39561349	Kluth et al.	1.41E − 06		1.79E − 02
BICF2G630212376	17	28929910	Kluth et al.	3.89E − 06	5.80E − 01	9.60E − 01
BICF2P28982	18	51795260	Kluth et al.	8.32E − 06		1.18E − 03
BICF2G630365393	23	48506877	Hayward et al.	2.28E − 05	7.91E − 04	4.70E − 03
BICF2S23410492	27	9400352	Kluth et al.	1.58E − 06	8.00E − 01	2.90E − 01
BICF2P507470	27	25549421	Kluth et al.	4.79E − 06		2.70E − 01
BICF2G630625485	29	23903463	Kluth et al.	5.50E − 06	4.30E − 01	9.40E − 01
BICF2G630405064	30	22647163	Hayward et al.	8.93E − 05	1.50E − 01	4.70E − 01
BICF2P113616	30	33816254	Hayward et al.	1.60E − 05		8.43E − 03
BICF2P1106247	30	37235914	Hayward et al.	7.25E − 06	2.34E − 02	2.47E − 02
BICF2G630740465	31	30836962	Kluth et al.	7.41E − 09		5.50E − 01
BICF2G630132623	37	27255309	Hayward et al.	1.54E − 04	5.00E − 02	
BICF2G63068103	38	21626523	Hayward et al.	8.22E − 05		1.37E − 02
BICF2G630473286	9	52717981	GWAS-AUS	2.23E − 05	1.90E − 01	7.74E − 01
BICF2G630233852	20	22031342	GWAS-AUS	1.97E − 05	1.04E − 08	3.81E − 07
BICF2G630233861	20	22045960	GWAS-AUS	8.44E − 06	1.40E − 08	2.60E − 07
BICF2P601097	20	22331987	GWAS-AUS	1.34E − 05	1.68E − 06	2.56E − 06
TIGRP2P402429	34	2084542	GWAS-AUS	3.27E − 05		
BICF2P1150303	34	235039	GWAS-AUS	1.70E − 05	3.18E − 05	2.71E − 03
BICF2S23521065	34	2397411	GWAS-AUS	3.25E − 05		
TIGRP2P402526	34	2419283	GWAS-AUS	1.04E − 05		
BICF2P1074499	36	30367554	GWAS-AUS	1.77E − 05		
BICF2P1371241	38	1449414	GWAS-AUS	1.94E − 05	7.30E − 04	4.98E − 05
BICF2P1152155	17	48910839	GWAS UK-USA (Quantitative)	7.32E − 05	5.07E − 04	1.10E − 02
BICF2G630202319	17	48926297	GWAS UK-USA (Quantitative)	1.36E − 04		
BICF2P522035	17	49139343	GWAS UK-USA (Quantitative)	9.08E − 05		
BICF2G630233852	20	22031342	GWAS UK-USA (Quantitative)	1.17E − 04	1.04E − 08	3.81E − 07
BICF2G630233888	20	22116909	GWAS UK-USA (Quantitative)	9.23E − 05	1.62E − 08	3.75E − 07
chr20_23130018	20	23130018	GWAS UK-USA (Quantitative)	8.02E − 05		
BICF2P950271	20	23148331	GWAS UK-USA (Quantitative)	3.50E − 05	1.10E − 05	1.69E − 03
BICF2P1295189	20	23191697	GWAS UK-USA (Quantitative)	2.44E − 05	6.86E − 06	1.32E − 03
BICF2P1315542	20	23218856	GWAS UK-USA (Quantitative)	1.21E − 04	1.06E − 05	2.85E − 03
BICF2P764018	34	666725	GWAS UK-USA (Quantitative)	1.31E − 04	9.10E − 05	1.36E − 03
BICF2P814549	1	67728530	GWAS UK-USA (CMH)	4.14E − 04		
BICF2S24318175	7	30788487	GWAS UK-USA (CMH)	3.38E − 04		
BICF2G63086882	7	78841165	GWAS UK-USA (CMH)	1.14E − 04	1.24E − 02	4.02E − 04
TIGRP2P272624_rs8804915	20	21661501	GWAS UK-USA (CMH)	3.72E − 04	5.12E − 06	1.36E − 05
BICF2G630233852	20	22031342	GWAS UK-USA (CMH)	2.74E − 04	1.04E − 08	3.81E − 07
BICF2G630233861	20	22045960	GWAS UK-USA (CMH)	3.11E − 04	1.40E − 08	2.60E − 07
BICF2G630233888	20	22116909	GWAS UK-USA (CMH)	1.19E − 04	1.62E − 08	3.75E − 07
BICF2G630234028	20	22399067	GWAS UK-USA (CMH)	3.95E − 04	1.78E − 05	1.71E − 05
BICF2P594756	24	35872204	GWAS UK-USA (CMH)	2.72E − 04		
BICF2P1033323	24	35906027	GWAS UK-USA (CMH)	2.72E − 04	1.37E − 02	7.06E − 04

Most significantly associated markers from previous GWAS analyses and GWAS performed in this study are listed on the left of the table. On the right are the results from the meta-analyses for these most significantly associated markers. Missing values represent variants that failed quality filtering. Variants are reported relative to canFam3.

### Putative functional variant detection

An approximately 300 kilobase region on CFA20 harbouring *MITF* was assessed using whole-genome sequence data of seven Australian Dalmatian dogs (5 bilateral deaf 2 hearing). The bioinformatic analysis identified a total of 1728 variants on CFA20 (UU_Cfam_GSD_1.0/canFam4 hereafter referred to as canFam4), including the most significantly associated markers previously identified by GWAS (Table S3). Manual inspection of the sequence alignments identified two SINE insertions; one at CFA20:22025737 (canFam4), previously identified by Karlsson et al.^26^, and one at CFA20:22168470 (canFam4), novel to this analysis. The second SINE (Fig. S2) potentially disrupts the 5’ UTR of two MITF transcripts (transcripts *MITF.10* and *MITF.14*). Of the identified variants, 1014 were single nucleotide variants (SNVs) and 714 were insertion/deletions (INDEL). After focusing on variants near or within *MITF* transcripts annotated as expressed in the canine in the canFam4 reference genome, and the exclusion of variants unique to dogs carrying the *MITF* retrotransposon as determined by heterozygosity for rs851676581 and rs851603213, a total of 26 variants (22 SNVs, two repeat-element insertions, and one length polymorphism) remained for consideration (Table 2). The SINE element insertion described by Karlsson et al.^26^ was monomorphic as was the newly described allele at rs853013202 CFA20:22022763–22022766AAA>T (canFam4), which is located in the PAX3/BRN2 binding site in the *MITF-M* promoter. Four of the five deaf Dalmatians were homozygous for the alternate allele across all 24 polymorphic variants assessed, while the two hearing Dalmatians were either heterozygous or homozygous for the reference allele. One deaf Dalmatian (Dog 80) shared genotypes of nine variants with the deaf dogs but shared genotypes of 15 variants with hearing dogs. The genomic region homozygous in all deaf dogs includes three variants located in the promotor of *MITF-M*, six variants in the 5’-UTRs of two dog-specific transcripts (*MITF.10* and *MITF.14*), and seven variants within the transcribed portions of long non-coding RNAs*.* A further nine variants identified in the vicinity of *MITF.1* first exon were poorly genotyped due to high GC content affecting genome sequence coverage. These are reported in Table S3 and are in the range CFA20: 22205233–22207639 (canFam4).

**Table 2:** Putative functional variants in the vicinity of the *MITF* gene discovered by alignment of whole genome sequencing data from seven Dalmatians to the canFam4 reference genome.

CFA	Location^ab^	Functional location	Position (bp) (canFam4)	Reference (canFam4)	Bilateral deaf					Hearing		Minor allele frequency	
					70	79	80	91	3360	71	131	MAF_722_^e^	MAF_590f._
20	RLOC_00009332.1	Transcript	21940904	C	**AA**	**AA**	**AA**	**AA**	**AA**	CA	CA	0.035	0.049
20	RLOC_00009332.1	Transcript	21940923	A	**AA**	**AA**	**AA**	**AA**	**AA**	TA	TA	0.423	0.341
20	RLOC_00009332.1	Transcript	21940972	C	**CC**	**CC**	**CC**	**0**	**CC**	TC	TC	0.379	0.327
20	RLOC_00009332.1	Transcript	21941161	A	**AA**	**AA**	**AA**	**AA**	**AA**	GA	GA	0.363	0.340
20	NM_001184968 (human)	3′ UTR	22020603	C	**TT**	**TT**	**TT**	**TT**	**TT**	CT	CT	0.004	0.008
20	rs853013202	PAX3/BRN-2 binding site	22022763–22022766	AAA	**TT**	**TT**	**TT**	**TT**	**TT**	**TT**	**TT**	N/A	N/A
20	MITF.8 and MITF.20 (MITFM)	Promoter (length polymorphism^c^)	22022796	31a^c^	**32b/32b**	**32b/32b**	32b/(13C7A2G12A)	**32b/32b**	**32b/32b**	32b/35b	32b/(13C7A2G12A)	N/A	N/A
20	MITF.8 and MITF.20 (MITFM)	Promoter	22022809	C	**CC**	**CC**	**CC**	**CC**	**CC**	CCTTTC	0	N/A	N/A
20	MITF.8 and MITF.20 (MITFM)	Promoter	22023329	G	**GG**	**GG**	**GG**	**GG**	**GG**	AG	AG	0.210	0.185
20	MITF	SINE- intronic^c^	22025737	DEL^c^	**INS/INS**	**INS/INS**	**INS/INS**	**INS/INS**	**INS/INS**	**INS/INS**	**INS/INS**	various	N/A
20	CF3 20:22031342 (ARRAY), MITF.10 and MITF.14	5′ UTR	22166843	T	**CC**	**CC**	TC	**CC**	**CC**	TC	TT	0.159	0.154
20	MITF.10 and MITF.14	5′ UTR	22167121	A	**CC**	**CC**	AC	**CC**	**CC**	AC	AC	0.158	0.153
20	MITF.10 and MITF.14	5′ UTR	22167597	A	**TT**	**TT**	AA	**TT**	**TT**	AT	AA	0.066	0.074
20	MITF.10 and MITF.14	SINE-5' UTR	22168470	DEL^d^	**INS**	**INS**	INS/DEL	**INS**	**INS**	INS/DEL	INS/DEL	N/A	N/A
20	MITF.10 and MITF.14	5′ UTR	22169237	A	**GG**	**GG**	AG	**GG**	**GG**	AG	AG	0.325	0.263
20	MITF.10 and MITF.14	5′ UTR	22169591	A	**GG**	**GG**	AG	**GG**	**GG**	AG	AA	0.327	0.265
20	MITF.10 and MITF.14	Promotor	22170942	C	**AA**	**AA**	CA	**AA**	**AA**	CA	CC	0.283	0.248
20	MITF.10 and MITF.14	Promotor	22171296	G	**AA**	**AA**	GA	**AA**	**AA**	GA	GG	0.287	0.249
20	CF3 20:22045960 (ARRAY)	Intron	22180699	T	**CC**	**CC**	TC	**CC**	**CC**	TC	TT	0.304	0.253
20	CF3 20:22116909 (ARRAY)	Intron, upstream	22250962	G	**AA**	**AA**	GA	**AA**	**AA**	GA	GG	0.306	0.339
20	RLOC_00009334.1 and MITF.24	Intron	22261165	G	**AA**	**AA**	GA	**AA**	**AA**	GA	GG	0.272	0.286
20	CF3 20:22369627 (ARRAY)	Intron, upstream	22302715	C	**CC**	**CC**	TC	**CC**	**CC**	TC	TT	0.241	0.229
20	MITF.24	Intron, upstream	22307828	A	**GG**	**GG**	AG	**GG**	**GG**	AG	AA	0.376	0.337
20	RLOC_00009335.1	Transcript	22341386	G	**AA**	**AA**	GA	**AA**	**AA**	GA	GG	0.188	0.161
20	RLOC_00009335.1,RLOC_00009335.2	Transcript	22363150	G	**GG**	**GG**	**GG**	**GG**	**GG**	GA	AG	0.265	0.191
20	RLOC_00009335.1,RLOC_00009335.2	Transcript	22374553	A	**AA**	**AA**	**AA**	**AA**	**AA**	CA	CA	0.19	0.141

Significant values are in [bold].

^a^Uppsala University GSD1.0 gene annotations.

^b^Non-dog RefSeq.

^c^Karlsson et al.^26^.

^d^Figure S2.

^e^Minor Allele Frequency (MAF) Plassais et al.^70^.

^f^MAF Jagannathan et al.^71^.

### Genome-wide re-analysis of publicly available data

A total of 304 Dalmatians sourced from the UK (N = 120) and the USA (N = 184) were subjected to a quantitative analysis using the first component of variance from multi-dimensional scaling as a covariate to account for population stratification, and a second GWAS applying the Cochran-Mantel Haenszel (CMH) test for stratified case–control data. After filtering, 125,209 variants and 304 dogs passed quality control and were included in subsequent analyses (Table S4). While none of the markers reached genome-wide significance in the genome-wide quantitative association analysis, six of the top 10 associated markers were located on CFA 20 (Table 1). All six markers were in close proximity to *MITF*. The remaining four markers were located on CFA17 (BICF2P1152155, BICF2G630202319, BICF2P522035) and CFA34 (BICF2P764018). The most significantly associated marker from the genome-wide quantitative association analysis was located at CFA20:23,191,697 (p = 2.4 × 10^–5^) (canFam3). Similarly, five of the top 10 markers from the genome-wide association analysis applying the CMH test were located on CFA20 (TIGRP2P272624_rs8804915, BICF2G630233852, BICF2G630233861, BICF2G630233888, BICF2G630234028), with the remaining markers on CFA1 (BICF2P814549), CFA7 (BICF2S24318175, BICF2G63086882), and CFA24 (BICF2P594756, BICF2P1033323) (Table 1). All associated markers on CFA20 were located within or near *MITF*, with two of the markers identified also amongst the top 10 associated markers in the quantitative analysis (CFA20:22031342 and CFA20:22116909) (canFam3). Interestingly, none of the top associated markers identified in previously published studies could be confirmed among the significant markers.

### Expanded meta-analysis

**Figure 1:**
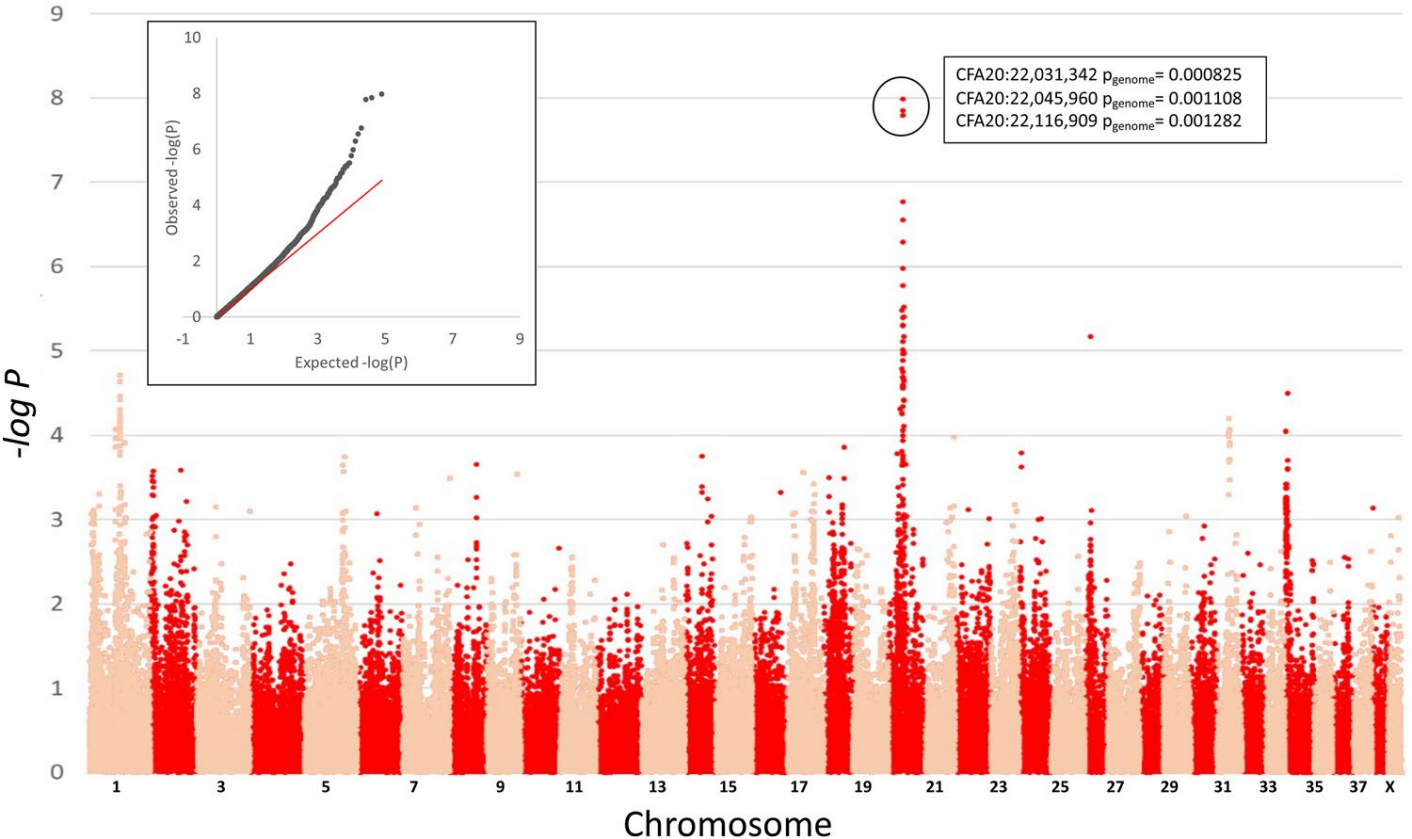
Genome-wide quantitative association meta-analysis of congenital sensorineural deafness in Dalmatian dogs. Negative log of probabilities for SNV markers based on quantitative trait association of congenital sensorineural deafness in 441 Dalmatian dogs sourced from three world continents. Animals were classified as either bilateral deaf, unilateral deaf or hearing based on recorded hearing testing results. Three markers associated with genome-wide significance are circled and their genome-wide significance reported. The Q–Q plot for the analysis is shown as an embedded image. The median Chi-squared value is (Lambda = 1.07551).

Merging the dataset of UK/US Dalmatians with the Australian dataset resulted in a total of 443 dogs with hearing phenotypes, representing three major global populations. Three dogs were represented in more than one cohort (Australian and UK/US), therefore one instance for each dog was coded as missing. After filtering, 79,316 variants remained for the linear association analysis and 92,972 variants for the CMH analysis. The genomic inflation factors were 1.07 and 1.32 respectively. The GWAS revealed a significant quantitative trait locus on CFA20, with all top 10 associated markers located on CFA20, spanning a genomic region of approximately 3 megabases (Table 3). The three most significantly associated SNVs were within or in close proximity to *MITF* (BICF2G630233852, P_raw_ = 1.04 × 10^−8^, P_genome_ = 0.0008; BICF2G630233861, P_raw_ = 1.04 × 10^−8^, P_genome_ = 0.001; and BICF2G630233888, P_raw_ = 1.62 × 10^−8^, P_genome_ = 0.001) (Fig. 1, Table S5) (canFam3). When the same dataset was subjected to the CMH test, four of the top 10 associated SNVs were located on CFA8 between 55,535,050 and 56,016,460 bp (canFam3). The remaining six SNVs were located on CFA20, covering the same genomic interval as identified in the quantitative analysis. Three of the top 10 SNVs were identified in both analyses, with all three SNVs located on CFA20 in close proximity to *MITF*.

**Table 3:** Top 10 associated markers from each meta-analyses using 441 Dalmatian dogs representing three major global populations.

SNP	CFA	Position (bp)	Analysis	Praw	Pgenome
BICF2G630232272	20	19929264	Meta-analysis (quantitative)	3.28E − 06	
BICF2G630233852	20	22031342	Meta-analysis (quantitative)	1.04E − 08	8.25E − 04
BICF2G630233861	20	22045960	Meta-analysis (quantitative)	1.40E − 08	1.11E − 03
BICF2G630233888	20	22116909	Meta-analysis (quantitative)	1.62E − 08	1.28E − 03
BICF2S23319907	20	22168603	Meta-analysis (quantitative)	5.14E − 07	4.07E − 02
BICF2S22962439	20	22179808	Meta-analysis (quantitative)	2.80E − 07	2.22E − 02
BICF2P1511957	20	22316076	Meta-analysis (quantitative)	1.05E − 06	
BICF2P601097	20	22331987	Meta-analysis (quantitative)	1.68E − 06	
BICF2P373083	20	22369627	Meta-analysis (quantitative)	1.71E − 07	1.35E − 02
BICF2P124796	20	22974211	Meta-analysis (quantitative)	3.05E − 06	
BICF2P288108	8	55535050	Meta-analysis (CMH)	3.78E − 06	
BICF2P1404889	8	55730559	Meta-analysis (CMH)	9.05E − 07	
BICF2P668242	8	55786946	Meta-analysis (CMH)	1.04E − 06	
BICF2S23510367	8	56016460	Meta-analysis (CMH)	2.32E − 07	2.16E − 02
BICF2G630233852	20	22031342	Meta-analysis (CMH)	3.81E − 07	3.55E − 02
BICF2G630233861	20	2,2045960	Meta-analysis (CMH)	2.60E − 07	2.42E − 02
BICF2G630233888	20	22116909	Meta-analysis (CMH)	3.75E − 07	3.49E − 02
BICF2S22962439	20	22179808	Meta-analysis (CMH)	1.10E − 05	
BICF2P601097	20	22331987	Meta-analysis (CMH)	2.56E − 06	
BICF2P373083	20	22369627	Meta-analysis (CMH)	1.09E − 05	

Variants are reported relative to canFam3.

None of the markers previously associated with deafness in Dalmatian dogs ranked amongst the highest associated SNVs in this meta-analysis (Table 1). Across all analyses performed in this study, the locus on CFA20 spanning the genomic region between BICF2G630233852 and BICF2G630233888 was consistently identified as significantly associated with deafness in Dalmatians, with one SNV consistently identified as the most significantly associated marker (BICF2G630233852, CFA20:22031342, P_raw_ = 1.04 × 10^−8^, P_genome_ = 0.0008).

### MITF risk haplotype analysis

**Figure 2:**
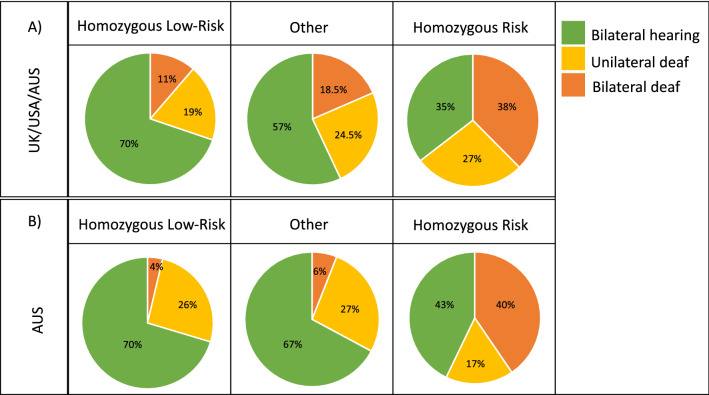
Deafness associated haplotype risk analysis. Deafness associated haplotype risk analysis using three genome-wide significant associated markers on CFA20 [BICF2G630233852T>C, BICF2G630233861T>C, BICF2G630233888G>A]. Animal were categorised as either homozygous risk haplotype, homozygous low-risk haplotype or other, with other including animals heterozygous for the risk/low-risk haplotype as well as other rare haplotype combinations. (**A**) shows the allocation of dogs (%) using the entire cohort of 442 dogs and (**B**) shows the allocation of dogs (%) using the Australian cohort of 139 dogs only. Animals classified as bilateral deaf are indicated in dark orange, unilateral deaf animals in yellow and hearing animals in green. Percentages of animals according to deafness status are included in the chart.

Genotypes for the three genome-wide significant associated markers on CFA20 (BICF2G630233852T>C, BICF2G630233861T>C, BICF2G630233888G>A) were extracted from all 442 Dalmatian dogs, resulting in 53 dogs homozygous for the canFam4 reference haplotype (TTG), 189 dogs homozygous for an alternative haplotype (CAA) and 200 dogs heterozygous (other) (Table S7). Of the 442 dogs, 114 were bilateral deaf, 110 were unilateral deaf and 218 were classified as hearing. When hearing status was assessed based on haplotype, a decreased risk of clinical deafness was observed in animals carrying the canFam4 reference low-risk haplotype (TTG) (Fig. 2A). The same trend was observed when the Australian cohort was analysed separately, with a tenfold increase of deafness in animals homozygous for the alternative haplotype (CCA) compared with animals homozygous for the low-risk haplotype (TTG) (Fig. 2B). While change in the proportion of unilateral deaf dogs was not significantly associated with the risk haplotype, the presence of at least one high-risk allele showed a general trend of reduced unilateral deafness with only 19% of unilateral deaf dogs homozygous for the low-risk haplotype, compared to 27% homozygous for the high risk haplotype (Fig. 2). Using quantitative phenotypes, the association P-values for the high-risk (CCA) and low-risk (TTG) haplotypes, are 2.09 × 10^−07^ and 1.73 × 10^−07^ respectively. The probability for the haplotype CCA under quantitative association analysis (bilateral deaf score 2, unilateral scored as 1.5, control scored as 1) was 3.02 × 10^−08^. Haplotype by phenotype combinations are tabulated for the individual geographic cohorts (Table S8).

### Association between pseudogene and deafness phenotype

**Figure 3:**
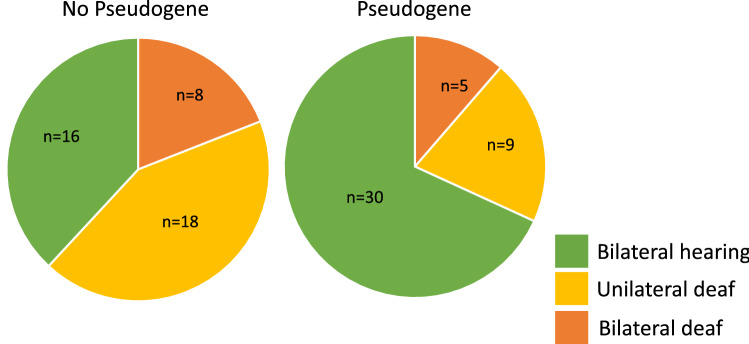
Relationship between *MITF* retrotransposon and deafness. Segregation of deafness phenotype according to presence or absence of *MITF* retrotransposon among animals genotyped using the Illumina 220 K array. Animals classified as bilateral deaf are indicated in dark orange, unilateral deaf animals in yellow and hearing animals in green.

A total of 14 markers in strong LD with the *MITF* retrotransposon were extracted from 89 animals genotyped on the 220 K genotyping array. The marker set included five markers on CFA19, seven markers on CFA20 and two markers on CFA32 (Table S6). While none of the 14 markers achieved genome-wide significance in the quantitative association, eight had a significant unadjusted quantitative association with CSD (P < 0.05). When hearing status was assessed against the presence or absence of the pseudogene, the proportion of hearing animals was increased among animals carrying the retrotransposon (Fig. 3).

## Discussion

White coat patterning caused by the *S* locus has long been established as a major risk factor for CSD in dogs^1,3,4,20^. In addition to the overall increase of white areas on the body, the risk of deafness increases in animals lacking facial pigmentation and with one or two blue irises^1,3,4,6,7,10,25,30^. Complex segregation analyses indicated that while CSD in Dalmatians is clearly heritable, the phenotype most likely follows a dichotomous or trichotomous mode of inheritance^12^. As a result, the *MITF* gene, which encodes the *S* locus, has been intensively studied but no causative mutation underlying CSD has been identified to date^26,38^. Using data from Dalmatian dogs representing three major global populations and applying multiple analyses enabled us to identify a significant association of CSD on CFA20, in the vicinity of *MITF*. More importantly, we identified a single, well-defined genome-wide significant risk haplotype for CSD in Dalmatian dogs.

The analysis of well characterised Australian Dalmatian dogs identified multiple array markers on CFA20 among the most associated markers with CSD. This finding was confirmed after combining all datasets available and re-analysing the entire Dalmatian data cohort with adjustment for stratification. This demonstrates that the association signal we have observed is consistent across populations and not unique to Australian-sourced animals. Utilising this population-level analysis enabled the identification of a strongly associated haplotype comprising three co-located markers (BICF2G630233852, BICF2G630233861, BICF2G630233888) that surround exon 1 of the *MITF-A* isoform (*MITF.1* Uppsala University GSD1.0)^42^ according to the canFam4 genome assembly. Animals homozygous for the non-reference haplotype had a significantly increased risk of deafness. The identified risk haplotype increases bilateral deafness risk with allele dosage in an additive manner (Fig. 2A) altering bilateral deafness frequency from 38% in animals homozygous for the risk allele to 11.7% in animals homozygous for the low-risk haplotype. By contrast, the effect on unilateral deafness is less dramatic with a reduction of only 8% from homozygous high risk to homozygous low risk (27% down to 19%).

A weak association at the *MITF* locus, and in fact the same region and haplotype as identified in our analyses, was previously identified by Stritzel et al.^38^. The same research group later confirmed the weak association at the *MITF* locus in a mapping experiment^35^. Interestingly, despite a larger cohort size, Hayward et al.^39^ did not identify *MITF* as a major risk locus for deafness in Dalmatians. It is likely that a combination of the reference assembly issues raised by Brancalion et al.^40^ acting to obscure signal in this vicinity, along with heterozygosity of the canFam3 reference dog through the critical region (as evidenced by all three markers of the major haplotype comprising SNV discovered in the boxer assembly), together hampered phasing of haplotypes^43^. Furthermore, accurate phenotyping is one of the most critical components when conducting genetic studies. Phenotyping errors due to different equipment, operators or sedation methods used, may result in animals being improperly allocated to affection status groups. While not always under the control of the researcher, these errors reduce the power to detect associations^44,45^. The signal at *MITF* identified as a leading association in the Australian data is most likely due to most animals being assessed by a single veterinary neurologist using high-end BAER testing equipment. Such testing is expensive, and many breeders opt to have testing carried out at local clinics that have access to other BAER testing equipment operated by less experienced practitioners. Sampling errors in phenotypes generated by the other equipment or other operators with less experience cannot be discounted as a reason for low signal detection across all studies.

As the canFam3 reference assembly includes the risk variant at BICF2G630233888, it is possible that the risk haplotype identified in our study may confer deafness risk in other breeds exhibiting extreme white coat phenotypes, and perhaps particularly, the boxer. This conclusion is supported by a recent study on deafness in Australian Cattle Dogs, which also identified an association between the *MITF* locus and deafness^46^. While the most associated SNV in Australian Cattle Dogs differs from the one identified in Dalmatians, the quantitative trait locus identified in these two breeds overlaps. It is possible that genetic selection for the low-risk genotype will be a useful selection metric to reduce ongoing deafness risk when BAER testing is difficult or expensive to obtain, but this remains to be tested in a planned multi-generational study.

In addition to issues with the reference assembly, accurate assessment of *MITF* has further been impaired by the recently identified *MITF* retrotransposon on the canine Y chromosome (*MITFY*)^40,41^. The accumulation of retrotransposon-specific variants has caused a sex based genotyping bias, which has likely hampered the identification of associations between *MITF* and CSD^41^. Considering that regulatory variants or epistatic interactions among remote loci or compound within-locus variant interactions may affect the complex phenotype in an unpredictable manner, previous analyses may have been underpowered to detect the signal with statistical significance. Despite the accumulation of variants and the introduction of a premature stop codon, there is evidence that the *MITF* retrocopy on the Y chromosome is expressed^41^. While the exact function of the resulting protein is yet to be investigated, it has been established that many miRNA binding sites are preserved in the expressed transcript. Investigations of the role of miRNAs in the regulation of melanogenesis have indicated that depending on coat colour, miRNA expression profiles differ significantly, and it has been established that the predicted targets of differentially expressed genes are involved in pigmentation^47–49^. This not only indicates a potential role of the *MITF* retrotransposon on pigmentation and pigmentation-related disorders but supports our finding that the presence of the retrotransposon potentially modifies the risk of CSD (Fig. S1).

When putative functional variants were assessed across the associated region on CFA20, we identified a haplotype that was shared by all sequenced (n = 5) bilateral deaf Dalmatians. The risk-haplotype variants include a previously described length polymorphism in the promotor region of *MITF-M* at CFA20:22,022,802 (canFam4). This length polymorphism has been identified as a key regulator for white spotting in dogs, with longer versions of the polymorphism resulting in reduced promotor activity^50^. We were surprised to observe that deaf animals with the high-risk haplotype have a shorter version of the length polymorphism allele 32b (11C7A2G12A) compared to hearing Dalmatians, which were allele 35b (11C10A2G12A) and the newly described variant of length allele 34 (13C7A2G12A) using the descriptive terminology of Karlsson et al.^26^ and Baranowska Körberg et al.^50^. The allele-length 34 variant was also observed in a heterozygous form in the discordant deaf Dalmatian (sample identifier = 80). Variants that were unique to sequenced Dalmatians that harboured the *MITF* retrotransposon (including individual 80) were excluded from our assessment of functional variants. Thus, it is possible that individual 80 is a compound heterozygote for deleterious mutations on two different haplotype backgrounds, and that an alternative risk mutation may occur on the length 34 haplotype background but was not captured due to our attempt to exclude retrotransposon variants. Segregation of two risk alleles on alternate risk haplotypes will increase the complexity of the locus and may play a role in suppressing GWAS mapping signals from the wider *MITF* locus. The presence of potential alternative risk variants on other haplotypes may be the reason that the protective haplotype was associated with stronger significance than the risk haplotype in the meta-analysis.

The extreme white spotting phenotype that is characteristic of all Dalmatians has been observed to result from significantly lower *MITF-M* promotor activity. This is likely affected by a SINE insertion polymorphism affecting *MITF-M* located at CFA20:22,025,737 (canFam4) first described by Karlsson et al.^26,50^. This variant was monomorphic (SINE present) among our sequenced dogs. However, low levels of bilateral deafness among animals with the low-risk haplotype suggest that while the extreme white encoded by the SINE element may partly affect deafness risk, the risk is modified by additional variants in the *MITF* region in a complex manner. New variants of interest include the newly identified variant at rs853013202 CFA20:22022763–22022766AAA>T (canFam4) and variants surrounding the MITF.16 isoform. While not polymorphic in the five Dalmatians analysed, the rs853013202 variant is located in the PAX3/BRN2 binding site of the *MITF-M* promotor. Both BRN2 and PAX3 influence *MITF* expression^51,52^, and it is plausible that this variant could influence the phenotypic expression of CSD. In contrast, variants surrounding exon 1 of the MITF.16 isoform were unique to deaf Dalmatians. This *MITF* isoform has only been described in the dog, and to fully understand the potential impact of these and other variants on melanocyte development, further functional analyses are required.

The *MITF-M* transcript is exclusively expressed in melanocytes derived from the neural crest and retinal pigment epithelium, and expression at these sites is pivotal for all melanogenic processes^53,54^. Melanocytes within the auditory and vestibular system of the inner ear play a pivotal role in maintaining auditory and vestibular function in mammals. Located in the stria vascularis, melanocytes are necessary for the generation of the endocochlear potential and decreases in this potential have been linked to age-related hearing loss in humans^55^. Reduced pigmentation of skin, coat and eye has been associated with inner ear disfunction in a range of mammals, including humans, dog, cat, and horse^1,4,56,57^. Based on the histological changes observed in the inner ear, deafness in the Dalmatian breed is most likely of the cochleo-saccular type^5,6,12,18–21^. Current results indicate that the degeneration of the stria vascularis in the cochleo-saccular type is caused by the absence of melanocytes in the stria vascularis^58–60^. Considering that our results suggest a protective function of the *MITFY* retrocopy, it is plausible that expressed *MITFY* transcripts recover at least part of the lost activity and stimulate melanocyte development.

As only incomplete data on iris colour was available for Dalmatians included in this analysis, iris colour was not considered in this study, although presence of blue or part-blue eyes was annotated on the USYD neurology samples. In these animals two observations of iris dilution were in pups with normal hearing. Previous research suggests that variants in *MITF* are not only associated with CSD in Dalmatians but also with blue irises, and that the presence of one or two blue irises increases the risk of deafness^16,38^. While studies from humans indicate that variants in *MITF* are not involved in determining normal eye colour, some disease-associated variants in *MITF* have been linked to blue irises^61^. The fact that not all disease associated *MITF* variants result in blue irises in humans could indicate that other variants in LD with the disease-causing *MITF* variant are responsible for blue irises, that depending on the location of the *MITF* variant, iris specific regulatory mechanisms are impacted, or that complex interactions among variants at the locus play a role.

The analysis reported in this study finds that variants in the vicinity of *MITF* increase deafness risk relative to wild-type alleles. We note that the penetrance of the risk haplotype for the deafness trait is modest with only 38% of dogs homozygous for the risk haplotype being phenotyped as clinically deaf in the combined data resource. Importantly, 12% of dogs homozygous for the low-risk haplotype were also tested as deaf (Table S8) with the majority of the 12% (five of six observations) being in the UK cohort of dogs. The UK cohort had an unusually low record of unilateral deaf dogs that may mean that some were classified along with bilateral deaf dogs as “deaf”. One deaf Australian dog that was homozygous TTG had other health issues (seizures) that may suggest a different disease phenotype. The combined CMH analysis (Table 3) detected genome-wide significant association on CFA8 (BICF2S23510367). These findings suggest that other loci or additional variants at this locus likely contribute to deafness in this breed.

Understanding the genetics of CSD in Dalmatian dogs has presented itself as a true challenge to numerous researchers. While many questions remain, we believe that results from this study open the door for genetic testing. In combination with currently applied selection criteria for breeding animals, selecting animals with the protective *MITF* haplotype will assist with reducing the number of deaf Dalmatians. Despite this new genetic tool, further studies investigating the underlying mechanisms and identifying functional variants will be crucial to eliminate CSD from this breed.

## Materials and methods

### Samples and genotyping

Blood or saliva samples of a total of 145 Dalmatians, comprising 86 hearing, 33 unilateral deaf and 26 bilateral deaf individuals were collected in Australia under University of Sydney Animal Ethics Committee (Approval numbers: N00/9-2009/3/5109; N00/10-2012/3/5837; and 2015/902) (Table S1). All experiments were performed in accordance with the recommendations of the Australian Code of the Care and Use of Animals for Scientific Purposes and reporting follows the recommendations in the ARRIVE guidelines. Genomic DNA was extracted from Performagene PG-100 buccal swabs (DNA Genotek, Canada) or EDTA whole-blood samples using the PureLink Genomic DNA Mini Kit (Invitrogen-Thermo Fisher Scientific, Waltham, Massachusetts, United States) following the manufacturers’ instructions. DNA quantity and quality was assessed using a NanoDrop2000 Spectrophotometer (Thermo Fisher, Waltham, Massachusetts, United States). DNA from blood spots applied to Whatman FTA cards was extracted by the genotyping provider (Neogen Inc, Nebraska USA). DNA samples of 139 individuals were genotyped using the Illumina Canine HD genotyping array (either 170 K or 220 K format) (Neogen Inc, Nebraska USA) and whole-genome sequences were generated for six animals (four bilateral deaf and two hearing) using 100 base paired-end Truseq libraries (Ramaciotti Centre for Functional Genomics) at 5–10× coverage on a HiSeq 2000. One bilateral deaf dog (Dog 3360) was sequenced on an Illumina XTen by the Kinghorn Cancer Centre using 100 base paired-end reads and a PCR-free library with approximate 30-fold genome coverage. Genotypes for array markers (canFam3) were extracted from alignments for the first six dogs with whole-genome sequencing data using Arraymaker^62^.

### Phenotyping

Hearing status of Australian Dalmatian dogs was determined with brainstem auditory evoked response (BAER) testing either performed at the University of Sydney Veterinary Teaching Hospital Sydney following routine veterinary protocols or BAER results provided by the owner (referred to as “owner-reported”). Eye colour was noted in dogs tested at the University of Sydney Veterinary Teaching Hospital Sydney but otherwise not explicitly recorded. Attributes of Australian dogs, including deafness status, phenotyping location, and genotyping or sequencing strategy are reported in Table S1. Characteristics of publicly available samples are as previously recorded^39^.

### Genome-wide association analysis

A GWAS of Australian data (N = 145) was conducted using quantitative association analysis in Plink (v1.90 64-bit)^63^. Data were coded to a quantitative phenotype score: control 1 (bilateral hearing), unilateral 1.5, deaf 2. Variants with a genotyping rate of < 0.1, strong deviation from Hardy–Weinberg expectation (p < 0.005), and a minor allele frequency (MAF) of < 0.1 were removed from further analysis. Genome-wide significance was ascertained by Bonferroni probability using the –adjust option in Plink^63^, with p-values < 0.05 considered as genome-wide significant. Top associated variants identified in this analysis were assessed against previously published associated markers.

### Putative functional variant detection

To assess for the presence of potentially functional variants, all variants were called in the associated region on CFA20 using whole-genome sequence data from seven Australian Dalmatian dogs. Paired sequencing reads were processed relative to the canFam4 reference assembly using standard pipelines including the use of the Burrows-Wheeler Aligner and samtools mpileup for variant detection^64–66^, to take advantage of improvements to the reference assembly throughout the *MITF* region^42^. Putative functional variants were called by visualising the regional variant call file (vcf) as a custom track on the UCSC browser and making use of the Variant Annotation Integrator tool^67,68^. Where necessary, the Integrative Genomics Viewer (IGV)^69^ was used to visualize variants and sequence alignments relative to the Uppsala GSD 1.0 gene annotations track on the University of California Santa Cruz genome browser for canFam4. Variants unique to dogs that carried the *MITF* retrotransposon (as ascertained by heterozygosity for missense variants rs851676581 and rs851603213) were excluded from further consideration^40^. Remaining variants were manually assessed for potential functional implications, concentrating on variants located near, or within functionally relevant *MITF* transcripts. Manual inspection in the IGV was used to identify putative repeat element insertions within the same regions. Where available, minor allele frequencies for the identified variants were assessed using two published variant resources representing 772 (two Dalmatian) and 590 (two Dalmatian) dogs and canids^70,71^ after first using the University of Santa Cruz genome browser liftover tool to convert the variant coordinates from canFam4 to canFam3.

### Genome-wide re-analysis of UK-USA data

Publicly available UK-USA Dalmatian genotyping array data (N = 304)^39^ were re-analysed using a quantitative analysis with the first component of multi-dimensional scaling fitted as a covariate to account for the geographic origins of the samples. Variants with a MAF < 0.05, a genotyping rate of < 0.1 and strong deviation from Hardy–Weinberg expectation (p < 0.005) were excluded from the analysis. Bilateral deaf dogs were coded as 2, unilateral as 1.5 and bilateral hearing as 1. Subsequently, the same dataset was subjected to a case–control CMH test for stratified data after using the cluster function in Plink to allocate samples to two predominantly geographical clusters. Deaf and unilateral dogs (N = 170) were coded as cases and bilateral hearing dogs were coded as controls (N = 134). Genome-wide significance was ascertained by Bonferroni probability using the adjusted option in Plink^63^, with adjusted p-values of < 0.05 considered genome-wide significant.

### Expanded meta-analysis

Publicly available genotyping data were merged with array data of 139 Australian Dalmatians with hearing phenotypes. A linear association analysis was performed using Plink^63^ excluding genotypes with a MAF < 0.05, a genotyping rate < 0.1, and strong deviation from Hardy–Weinberg expectation (p < 0.005). Data were corrected for population stratification by fitting the first and second principal components (C1 and C2) from multi-dimensional scaling analysis as co-variates in the analysis. All data were recoded to a quantitative phenotype (scores: control 1, unilateral 1.5, deaf 2). Using identical filtering criteria for variants, the same expanded cohort of dogs was subjected to a case–control analysis using a CMH test for stratified data. Deaf and unilateral dogs (N = 224) were coded as cases and hearing dogs as controls (N = 216). The cluster function in Plink^63^ was used to allocate samples to three predominantly geographical clusters. Top associated variants were assessed against previously published associated markers and associated markers from the Australian cohort and vice versa.

### Deafness associated risk haplotype analysis

The three most associated markers on CFA20 (BICF2G630233861, BICF2G630233888, BICF2G630233852) were used to perform a haplotype risk analysis. Marker information and corresponding haplotypes were extracted from arrays for all 443 dogs using Plink and a quantitative haplotype risk analysis performed. Deaf animals were scored as 2, unilateral deaf as 1.5 and hearing as 1. Animal hearing status was assessed against haplotypes with animals grouped as either homozygous for the reference haplotype, homozygous for the alternative haplotype, or heterozygous. The cohort of 139 Australian Dalmatians was analysed separately.

### Deafness association of pseudogene

Dogs were assessed for the presence of the *MITF* retrotransposon as described before^40^. Information on sex was not available for all dogs included in this study, therefore markers in strong LD with the *MITF* retrotransposon were extracted and a quantitative association analysis was performed using Plink^63^ to assess the potential involvement of the *MITF* pseudogene on hearing. As relevant markers are only present on the 220 K genotyping array, dogs genotyped on the 170 K array were excluded from this analysis.

### Ethics declaration

Recommendations from the Australian Code of the Care and Use of Animals for Scientific Purposes were strictly adhered to throughout this study. Research was conducted at The University of Sydney, under the Animal Ethics Committee approval no.: Approval numbers: N00/9-2009/3/5109; N00/10-2012/3/5837; and 2015/902. BAER testing was performed at The University Veterinary Teaching Hospital Camperdown following routine veterinary protocols. Reporting in this manuscript is in accordance with the ARRIVE guidelines.

## Supplementary Information

Supplementary Legends.

Supplementary Figure S1.

Supplementary Figure S2.

Supplementary Table S1.

Supplementary Table S2.

Supplementary Table S3.

Supplementary Table S4.

Supplementary Table S5.

Supplementary Table S6.

Supplementary Table S7.

Supplementary Table S8.

## Data Availability

Whole genome sequencing data connected with this project are available via the European Nucleotide Archive https://www.ebi.ac.uk/ena/browser/view/PRJEB53145.
