# Supplementary material for: De-novo and genome-wide meta-analyses identify a risk haplotype for congenital sensorineural deafness in Dalmatian dogs
Source: Sci Rep. 2022 Sep 14;12:15439. doi: 10.1038/s41598-022-19535-4 (PMC9474838; doi:10.1038/s41598-022-19535-4)
Supplement: Supplementary file 1 — Supplementary Legends. [file 41598_2022_19535_MOESM1_ESM.docx]

**Supplementary Table information**

Supplementary Table S1: Sample information of Dalmatian dogs included in the study. In addition to information on hearing status, information on gender, source, and source of genotyping (170: 170K array; 220: 220K array; WGS: whole-genome sequencing) have been included. For USYD neurology samples, presence of blue or part-blue eye is noted.

Supplementary Table S2: Genome-wide quantitative association analysis based on Australian cohort (the top 200 markers taken forward are shown at the top of the table, thereafter markers are in genome order). Coordinates are reported relative to canFam3.

Supplementary Table S3: Variant call format file across the associated locus on CFA20 using whole-genome sequence data of seven well characterised Australian Dalmatian dogs. Coordinates are reported relative to canFam4.

Supplementary Table S4: Genome-wide quantitative association analysis based on USA-UK cohort analyses (linear) and (Cochran-Mantel-Haenszel (CMH)). Coordinates are reported relative to canFam3.

Supplementary Table S5: Meta-analysis of merged Australian and Hayward et al data in association mapping analyses (linear) and CMH (Cochran-Mantel-Haenszel (CMH)) Coordinates are reported relative to canFam3.

Supplementary Table S6: Quantitative association of 14 variants in linkage disequilibrium with *MITF* retrotransposon with congenital sensorineural deafness (Coordinates relative to canFam3)

Supplementary Table S7: Genotypes of the three most associated markers on CFA20 (BICF2G630233861, BICF2G630233888, BICF2G630233852) and the hearing status of dogs used for assessing risk haplotypes.

Supplementary Table S8: Haplotype distribution at the associated locus on CFA20 by geographical cohort and phenotype.

**Supplementary Figure information**

Supplementary Figure S1: Superimposed images of the canFam4 region CFA20:22,167,528-22,169,415 in the UCSC Browser and the Integrative Genomics view showing the location of a SINE insertion in 5’ UTR for *MITF* isoforms *MITF.10* and *MITF.14*

Supplementary Figure S2: Visualisation of the newly discovered SINE insertion at CFA20:221684670, including representative reads taken from one of the Australian Dalmatian dogs with whole-genome sequence.
